# Comparative Study of Real-Time PCR (TaqMan Probe and Sybr Green), Serological Techniques (ELISA, IFA and DAT) and Clinical Signs Evaluation, for the Diagnosis of Canine Leishmaniasis in Experimentally Infected Dogs

**DOI:** 10.3390/microorganisms9122627

**Published:** 2021-12-20

**Authors:** María Paz Peris, Adriana Esteban-Gil, Paula Ortega-Hernández, Mariano Morales, Nabil Halaihel, Juan Antonio Castillo

**Affiliations:** Animal Pathology Department, Veterinary Faculty, University of Zaragoza, 50013 Zaragoza, Spain; adriesgil@gmail.com (A.E.-G.); paulish_zgz@hotmail.com (P.O.-H.); mjma1962@gmail.com (M.M.); halaihen@yahoo.com (N.H.); jacasti@unizar.es (J.A.C.)

**Keywords:** *Leishmania* diagnosis, canine leishmaniasis, real time PCR, serological evaluation

## Abstract

Canine leishmaniasis (CanL) diagnosis is not fully resolved. Currently, two specific methodologies are in continuous development, the detection of the parasite DNA or RNA in target organs and the detection of specific antibodies against *Leishmania* sp. For a correct diagnosis, it has been shown that the joint use of this type of test is necessary. In this work, a Sybr Green and a TaqMan Probe based on real time PCRs (qPCR) was performed for the detection of *Leishmania* sp. in order to correlate the results with clinicopathological and serological evaluations (IFA, ELISA and DAT) to propose an optimal biological sample to be used to detect the parasite in both early and late stages of the infection. A total of four samples were processed: conjunctival swabs, popliteal lymph node aspirates, bone marrow aspirates, and peripheral blood from experimentally infected dogs belonging to a larger study. Our results indicated that a single non-invasive sample (conjunctival swab) and the application of both types of qPCR would be reliable for determining *Leishmania* infection as well as the disease stage in dogs, thus avoiding bone marrow, lymph node aspirate or blood samples collection.

## 1. Introduction

Leishmaniasis is one of the most important neglected tropical diseases according to WHO [1]. The parasite *Leishmania infantum* has been identified as the main etiologic agent of canine visceral leishmaniasis, (CVL) which is a major global zoonosis that is potentially fatal to humans and dogs [2].

Several organs can be affected during the progression of the disease, including the skin, kidneys, spleen, liver, and eyes, and is characterized by a range of associated clinical signs such as skin lesions, generalized lymphadenopathy, weight loss, muscle atrophy, intolerance to exercise, loss of appetite, lethargy, splenomegaly, polyuria, polydipsia, ocular lesion, epistaxis, onychogryphosis, anemia, vomiting, and diarrhea [3].

Diagnosis is made considering the epidemiological origin and the set of clinical signs presented by the dog [4]. However, there are many asymptomatic dogs without pathognomonic clinical signs, so laboratory diagnosis confirmation is needed. All the parasitological, immunological, and molecular techniques available for diagnosis are important and need to be interpreted according to their benefits and limitations and are used individually or in combination [5].

Specific detection of antibodies against *Leishmania* sp., is preferably performed using quantitative serological techniques like indirect immunofluorescent assay (IFA), the direct agglutination test (DAT) and the enzyme-linked immunosorbent assay (ELISA). However, serological tests present important limitations, such as cross-reactions with *Trypanosoma* parasites, cutaneous leishmaniasis species, and other hemoparasites [6,7], as well as false negative results in dubious reactions [8].

Molecular techniques (conventional, nested and real time PCR) have high sensitivity and specificity, and are currently part of the veterinary diagnostic routine, which are especially useful for follow-up and may be performed on various biological samples, such as peripheral blood, bone marrow aspirate or lymph nodes, skin fragments, and others [9,10]. It is important to highlight that information provided by PCR should not be separated from the data obtained from clinicopathological and serological evaluations [4].

In this work, a Sybr Green and a TaqMan probe-based real time PCRs (qPCR) was used for the detection of *Leishmania* sp. in different samples from experimentally infected dogs: conjunctival swabs, popliteal lymph node aspirates, bone marrow aspirates and peripheral blood were performed. The main objectives were to correlate the data obtained from both qPCR methods with clinicopathological and serological evaluations to propose an optimal diagnostic tool to be used to detect the parasite in different stages of the infection.

## 2. Materials and Methods

### 2.1. Animals

Thirty-three (15 females and 18 males), healthy, intact, 12 to18-month-old, around 14 Kg beagle dogs derived from a larger experiment were used for this study. Thirty of them were experimentally infected via cephalic vein with 1 mL containing 10^8^ promastigotes per mL of *L. infantum* (MCAN/ES/Z002) obtained from a naturally infected dog as previously described methods [11]. The three remaining dogs were kept as uninfected controls.

All animals were periodically examined to determine their health status and were housed and maintained at optimal conditions.

All applicable international, national, and/or institutional guidelines for the care and use of animals were followed (Spanish Policy for Animal Protection RD53/2013, which meets the European Union Directive 2010/63 on the protection of animals used for experimental and other scientific purposes). All experimental practices were approved by the Ethics Committee for Animal Experiments from the University of Zaragoza (Project license PI28/14, date of approval: 4 June 2014).

### 2.2. Samples Collection

Twelve months after experimental infection, four different samples were collected from dogs previously sedated with 0.02 mg/kg IV of medetomidine (Domitor**^®^**, Orion Pharma, Spoo, Finland): (i) bone marrow (100–200 µL) by sternal puncture and aspiration into EDTA coated tubes; (ii) popliteal lymph node using fine needle aspiration and homogenized in 100 µL of sterile physiological saline serum; (iii) 2 different blood samples using fine-needle aspiration from cephalic vein: 1 mL deposited in normal tubes for obtaining serum, and another 1mL in EDTA coated tubes; (iv) conjunctival swab deposited in a sterile 1.5 mL tube.

### 2.3. Clinical Signs Assessment

One year after the experimental infection, no animals with azotemia or high proteinuria values were found, so the clinical assessment was performed in dogs by observing the severity of signs due to *Leishmania* infection. The absence/presence and intensity of every clinical sign was scored from 0 to 11 as previously described [12], with some modifications: (a) size of lymph nodes (0: normal; 1: enlargement); (b) skin involvement (0: normal; 1: slight scaling and/or alopecia; 2: severe alopecia and/or lesions); (c) weight loss (0: absence; 1: moderate <20%; 2: severe >20%); (d) ocular lesions (0: absence; 1: moderate; 2: severe); (e) onychogryphosis (0: absence; 1: presence); (f) muscle atrophy (0: absence; 1: presence); (g) pale mucous membranes (0: absence; 1: presence); (h) splenomegaly to palpation (0: absence; 1: presence). The final score was the sum of all numerical sign evaluation for an animal. Data was expressed as clinical signs score (CSS) and divided into three groups that reflects the disease severity. Group 1: dogs with CSS ≤2 indicating normal animals or with low clinical manifestations; Group 2: dogs with CSS between 3 and 6 for animals with moderate clinical manifestations; Group 3: dogs with CSS between 7 and 11 for animals with severe clinical manifestations.

### 2.4. Serological Analysis

Different serological techniques were used to evaluate *Leishmania* antibody concentrations in serum: a commercial ELISA test, an *in-house* DAT, and an *in-house* IFA.

ELISA was performed with a commercial kit according to the manufacturer’s instructions with a sensitivity of 92.5% and a specificity of 100% [13] (CIVTEST CANIS LEISHMANIA 192, Hipra Laboratories S.A., Gerona, Spain). The sera were considered negative (calculated value (CV) < 0.9), doubtful (0.9 < CV < 1.1) and positive (CV > 1.1).

In-house DAT and IFA were performed in the Parasitology Laboratory of the Veterinary Faculty of Zaragoza.

DAT antigen was obtained according to Easy-DAT antigen method previously described [14]. Then, samples were diluted in physiological saline (0.9% NaCl) containing 0.78% ß-mercaptoethanol and 0.2% gelatin. Two-fold dilution series of the sera were made in a V-shaped microliter plate, starting at a dilution of 1:100 and up to 1:6400. Fifty microliters (5 × 10^7^ parasites per ml) of Easy-DAT antigen (concentration was added to each well containing 50 µL of diluted serum). After two minutes of gentle shaking on a leveled platform, the plate was then covered with a lid and incubated at room temperature in dark for 18 h. A negative result was considered when a blue button was formed in the well. Two independent blind readings were performed by two technicians. A sample was considered negative when DAT dilution was ≤1:200, doubtful when dilution was 1:400 and positive when dilution was ≥1:800.

The in-house IFA was performed as described in the Manual of the World Organization for Animal Health [15]. The antigen used was obtained from the Parasitology Laboratory and had a concentration of 1 × 10^5^ of promastigotes of *L. infantum* in formalin per mL. The serum samples were two-fold diluted starting at a dilution of 1:20 in PBS (0.1 M phosphate, 0.33 M NaCl, pH 7.2) and up to 1:640. Ten microliters of each diluted serum was placed in one of the 12 wells of the slides and incubated in a humid chamber at 37°C for 30 min. Slides were washed in PBS and then incubated for 30 min at 37 °C with fluorescent rabbit anti-dog FITC-conjugated (Megacor Diagnostik, Hoerbranz, Austria). Slides were washed and air dried. Finally, the samples were observed under immunofluorescent microscope by two laboratory technicians. A sample was considered as negative when the fluorescent signal was absent or only observed at the 1:20 dilution, doubtful up till 1:40 dilution and positive once 1:80 or higher dilution.

### 2.5. Real-Time Quantitative Polymerase Chain Reaction (qPCR) Evaluation

Genomic DNA was extracted from the defrosted samples from −80 °C using a commercial Speedtools DNA extraction kit (Biotools B&M Labs S.A, Madrid, Spain). DNA from bone marrow aspirates (100 µL), popliteal lymph node aspirates (100 µL) and peripheral blood (100 µL) was obtained as per the manufacturer’s instructions. For the conjunctival swabs the protocol was modified. Each swab was suspended in 200 µL of lysis buffer with 25 µL of proteinase K and incubated at 70 °C for 1 h. Then, swabs were centrifuged in a preclearing filter before the whole volume was deposited in the extraction columns to follow next steps as described by the manufacturer. Extracted DNA was eluted in elution buffer (100 µL) and stored at −20 °C until further use.

Two qPCR methods were performed for the molecular evaluation: a TaqMan Probe based qPCR and an intercalating dye (Sybr Green) qPCR assay. Both amplifying specific sequences of the minicircle kinetoplast DNA (kDNA) (Table 1).

A probe-based qPCR assay (TaqMan-qPCR) was designed through a PrimerQuest™ Tool from Integrated DNA Technologies (Coralville, IA, USA). The technique was performed in a final volume of 20 µL containing 10 µL TaqMan master mixture (Quantimix Easy Probes, Biotools B&M Labs S.A, Madrid, Spain) and 2.5 µL of DNA template. The forward and reverse primer concentrations were adjusted to 0.4 μmol L^−1^ and a 0.3 μmol L^−1^ for the probe.

The Sybr Green based qPCR assay (Sybr-qPCR) was designed to be amplified by the fluorochrome known as Sybr Green and was performed according to previously described methods [16]. Each reaction was carried out in a final volume of 20 μL containing 10 μL of Sybr Green master mix (GoTaq**^®^** Hot Start Green Master Mix 2×, Promega, Madison, WI, USA) and 2.5 μL of DNA template. The forward and reverse primer concentration was adjusted to 0.4 μmol L^−1^ each.

**Figure 1:**
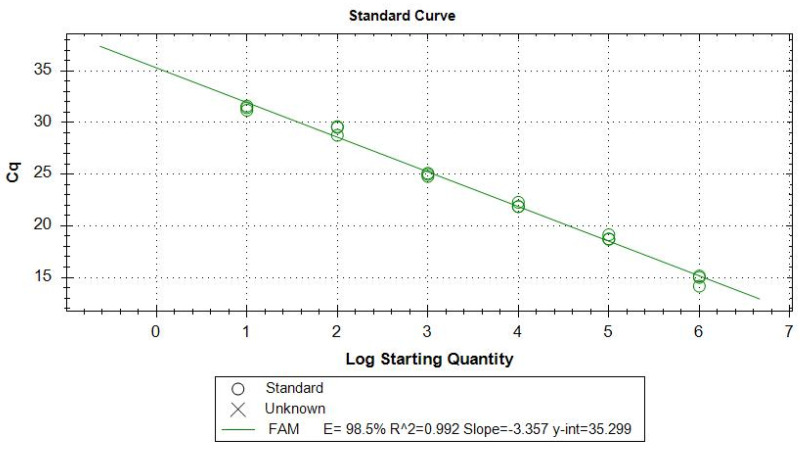
Standard curve for TaqMan-qPCR from known cultured promastigote concentrations.

To calculate the efficiency of the TaqMan-qPCR, a standard curve was performed from a concentration of 2 × 10^6^ promastigotes per mL of pure culture previously quantified by Fuchs-Rosenthal chamber, then achieving six logarithmic point dilutions. The linear equation resulting from the standard curve allowed for the defining of the efficiency of amplification. E = 98.5% with a correlation curve fit value of R^2^ = 0.99 (Figure 1).

All amplifications were conducted in a CFX Real-Time PCR System (Bio-Rad Laboratories Inc., Richmond, WA, USA) with an initial incubation at 95 °C for 10 min, followed by 44 cycles of 95 °C for 5 s (denaturation), 55 °C for 40 s (annealing, amplification and acquisition of fluorescence).

Each qPCR run included a negative control, a positive control, and a separate reaction for β-actin DNA copies as internal control [17]. Each determination was made by triplicate.

For statistical analyses purposes when no amplification was detected, a Ct value of 40 was granted.

### 2.6. Statistical Analysis

The Shapiro-Wilk test was performed to assess the normality of data. For non-normally distributed data, the non-parametric Kruskal-Wallis test was used to determine any statistically significant differences between groups. Dunn’s post hoc test adjusted by Bonferroni was used for pair-wise group comparison. IFA and DAT titers were expressed as respective logarithms for statistical evaluation. The correlation between the parameters was studied by Spearman’s rank correlation (rs) when considering non-parametric data and the interpretation was: when rs = 0.00–0.10 = negligible correlation; 0.10–0.39 = weak correlation; 0.40–0.69 = moderate correlation; 0.70–0.89 = strong correlation and 0.90–1 = very strong correlation. Statistical analyses were performed with SPSS v24 (IBM Corporation, Armonk, NY, USA), and statistical significance was set at α = 0.05.

## 3. Results

### 3.1. Real Time PCR Results

Out of the 30 animals, TaqMan-qPCR showed positive amplification in 23 bone marrow aspirates (76%), 26 in popliteal lymph node aspirates (86%), 4 in peripheral blood (13%) and 22 in conjunctival swab (73%). Sybr-qPCR showed *Leishmania* DNA presence in all biological samples from each infected dog in the study.

In both molecular techniques higher Ct values were observed in blood sample, middle Ct values in conjunctival swab and the lowest Ct values in bone marrow and popliteal lymph node aspirates (Table 2).

Correlation between both molecular techniques was very strong for bone marrow (rs = 0.94; *p* < 0.001) and conjunctival swabs (rs = 0.918; *p* < 0.001), strong for popliteal lymph node aspirate (rs = 0.74; *p* < 0.001), and moderate for peripheral blood (rs = 0.50; *p* = 0.004).

When TaqMan-qPCR results were considered, bone marrow showed moderate correlation with conjunctival swab (rs = 0.46; *p* = 0.009) and with popliteal lymph node (rs = 0.47; *p* = 0.008). Whereas, conjunctival swab and popliteal lymph node showed weak correlation (rs = 0.36; *p* = 0.049).

Regarding Sybr-qPCR, Ct results showed moderate correlation between bone marrow and popliteal lymph node aspirates (rs = 0.49; *p* = 0.006) and weak correlation between bone marrow and conjunctival swab (rs = 0.38; *p* = 0.041).

Peripheral blood sample showed non-significant correlation with the rest biological samples studied.

### 3.2. Clinical Evaluation and Correlation with qPCR Results

According to clinical signs classification, 10/30 animals (33.3%) were included in Group 1, 13/30 (43.3%) in Group 2 and 7/30 (23.3%) in Group 3.

TaqMan-qPCR evaluation showed that bone marrow, peripheral blood and conjunctival swab results differed significantly among clinical signs groups (*p* = 0.025, *p* = 0.029 and 0.041, respectively). However, only conjunctival swab Ct median values increased progressively from Group 3 to Group 1, showing good concordance between clinical signs and Leishmania-DNA load. Unexpectedly, bone marrow and popliteal lymph node samples Ct median did not show this tendency (Table 3).

Statistical analyses showed that Sybr-qPCR in bone marrow and conjunctival swabs medians differed significantly among clinical signs groups (*p* = 0.016 and *p* = 0.048, respectively). Furthermore, conjunctival swabs Ct median values increased progressively from Group 3 to Group 1, showing good concordance between clinical sings and Leishmania-DNA load. However, bone marrow, popliteal lymph node and peripheral blood value increments were not in concordance with clinical signs groups (Table 4).

### 3.3. Serological Evaluation and Correlation with qPCR Results

According to DAT results, nine out of 30 animals (30%) were positive, 11 (36.7%) were doubtful and 10 (33.3%) were negative. IFA detected 24 (80%) positives, five (16.7%) doubtful and one (3.3%) negative. Finally, ELISA showed 24 (80%) positives and 6 (20%) negatives, presenting no doubtful results.

Correlation between serological techniques was strong between DAT and IFA (rs = 0.78; *p* < 0.001) and between ELISA and IFA (rs = 0.73; *p* < 0.001). Moderate correlation was obtained between DAT and ELISA (rs = 0.59; *p* = 0.001).

Regarding the serological and TaqMan-qPCR evaluation relationship, it was observed that bone marrow median Ct values differed significantly among serological groups from all serological techniques. Conjunctival swab median Ct values differed significantly between positive and negative ELISA results. The highest Ct values corresponded to lowest antibody levels evaluated by IFA and ELISA in all processed samples. Furthermore, the Ct median values increased progressively from positive to negative antibody levels. However, DAT technique results were not in concordance with the parasite load increment except for conjunctival swab samples (Table 5).

Bone marrow, popliteal lymph node and conjunctival swab Sybr-qPCR results showed that positive animals against *L. infantum* antibodies (detectable by DAT, IFA, and ELISA) showed lower median Ct values than those that did not develop antibodies. However, this tendency was not observed with peripheral blood. Statistical analyses detected significant differences between bone marrow Ct median values and serological results. ELISA values increased progressively to *Leishmania* DNA levels in all biological samples. (Table 6).

## 4. Discussion

This study correlates the clinical signs and the antibody titers with the parasite load in different biological samples in an experimental model for CVL. Bone marrow is considered the target tissue for the invasion and multiplication of the parasite [18], and therefore it is usually the tissue where the highest concentration of *L. infantum* kDNA is detected [19]. However, in recent studies where the skin, lymph node, bone marrow and conjunctival swab were evaluated, they concluded that the skin [20] and the conjunctival swab [21] were the tissues that showed the highest rate of positivity. In our study, the highest parasite load corresponded to the bone marrow, followed by the lymph node, conjunctival swabs and finally the peripheral blood. Our results showed once more that blood is not the sample of choice for the molecular diagnosis of *Leishmania* [22], possibly due to inhibitors that reduce the sensitivity of the technique [23]. In addition, blood acts as a transport system for the protozoan and not as a reservoir organ [18].

When we compare the two qPCR techniques used in this work, we observe that the Sybr-qPCR technique has a much higher sensitivity since it detects parasite kDNA in the totality of the experimentally infected animals’ samples. However, TaqMan-qPCR detection percentages were 76% for bone marrow, 86% for lymph node, 13% for blood and 73% for conjunctival swab samples, respectively. Comparing the type of sample, we observed a good correlation between the conjunctival swab and the bone marrow (TaqMan-qPCR: rs = 0.46; Sybr-qPCR: rs = 0.38). This leads us to emphasize that a minimally invasive sample (conjunctival swab), easy to obtain without the need for animal sedation, would reproduce similar results compared to the target tissue (bone marrow).

Recently, studies have been carried out relating the clinic with the parasitic load in different tissues. For example, Chagas et al. [20], concluded that the skin and lymph node are the target tissues to monitor infected dogs with different clinical states ahead of the bone marrow and conjunctival swab. Aschar et al. [24] also propose the lymph node ahead of the conjunctiva swab, skin, blood, and oral cavity. However, other works [25] observed no significant differences with respect to canine clinical manifestation and the parasite loads detected in the blood, skin, and spleen samples. Our data yielded similar results to Ferreira et al. [26], where they detected a greater parasitic load in symptomatic dogs compared to asymptomatic, both in conjunctiva swab and bone marrow. Regarding the skin, no differences in amastigotes load were detected between the groups. In our work no skin sample was taken so we cannot assess the usefulness of this kind of samples. Furthermore, it should be noted that the conjunctival swab sample (both in TaqMan-qPCR and Sybr-qPCR) was the one that showed the best relationship with the three clinical groups, observing a progressive increase in the load depending on the severity of the clinical signs.

Renal parameters were not included in our clinical classification because none of the animals showed azotemia or significant proteinuria values (data not shown). This is in concordance with other published dealing with *Leishmania* infections. For instance, Fernández-Cotrina et al. [27] concluded that 12 months after experimental *Leishmania infatum* infection, the urea, creatinine and ALT levels in serum were within the normal physiological range in all animals. It is worth mentioning that similar clinical classification has been already used in *Leishmania* natural infection studies [20,24,25]. Besides, most authors affirm that renal alterations occur at least one-year post infection [28]. It would be reasonable to include renal parameter data in clinical classification, if the study was for longer than 18 months.

Serological methods such as IFA, DAT and ELISA are the most widely used diagnostic techniques in clinical and research studies of CVL. Some authors [29,30] consider IFA as the reference technique for the serological diagnosis due to its good sensitivity and specificity. However, according to our results, ELISA showed the same number of positive animals as IFA, with no doubtful results, which generally leads to repeat the test. In a study carried out by Solano-Gallego et al. [13], three commercial ELISA techniques, a rapid test and an *in-house* IFA were compared in naturally infected dogs, and the most sensitive technique was the same commercial ELISA than the one employed in this study. Several authors recommend molecular diagnosis by qPCR, in endemic areas, especially when dogs show compatible clinical signs with doubtful serological results [10,30].

Our IFA and ELISA serological results show a good relationship with the parasite load in all biological samples. However, DAT results were inconsistent, since the highest parasite load in lymph node, bone marrow and blood occurred when serology was doubtful. Both TaqMan-qPCR and Sybr-qPCR from bone marrow samples were statistically in concordance with the categorical groups determined by serology test, DAT, IFA, and ELISA. Likewise, conjunctival swab TaqMan qPCR results were comparable to the results for antibodies assessed by ELISA.

Considering the results of this work, the use of both molecular detection techniques is proposed using conjunctival swabs. Sybr-qPCR would be useful for detecting the presence of the parasite since it has proven to be very sensitive detecting *L. infantum* kDNA in all types of samples from the infected animals. TaqMan-qPCR, although it shows less sensitivity, has shown great capacity to differentiate animals in different clinical stages with the conjunctival swab sample. Interestingly, the conjunctival swab TaqMan-qPCR median Ct values were in concordance with serological ELISA results.

## 5. Conclusions

Our results indicate that a single non-invasive sample (conjunctival swab), by applying both types of qPCR (Sybr Green and TaqMan probe) would be reliable for determining *Leishmania* infection as well as the disease stage in dogs, thus avoiding bone marrow, lymph node aspirate or blood sample collection.

## Figures and Tables

**Table 1:** Primers and probe used for *Leishmania* detection.

Name	Sequences	Lenth (nt)	Reference
Leish-F	5′-CAAACCTATGCTCACTATC-3′	96	This article
Leish-R	5′-GGTATGGGTATTCTTTATGG-3′		
Leish-Probe	5′-FAM-CAACCACCACCATCAAATCC-3′-IABkFQ		
Jw11-F	5′-CCTATTTTACACCAACCCCCAGT-3′	116	[16]
Jw12-R	5′-GGGTAGGGGCGTTCTGCGAAA-3′		

nt: nucleotide.

**Table 2:** Median, minimum, and maximum Ct values in the different biological samples studied.

	TaqMan-qPCR	Sybr-qPCR
Bone marrow	25.1 (17.2-ND)	24.8 (17.7–33.9)
Popliteal lymph node	25 (17.5-ND)	25.1 (19.1–33.6)
Peripheral blood	ND (31.3-ND)	33.3 (27.4–35.6)
Conjunctival swabs	29.6 (24-ND)	30.8 (24.1–35.3)

ND: Ct ≥ 40.

**Table 3:** TaqMan-qPCR results in the different biological samples studied according to clinical signs classification.

	Bone Marrow ^1^	Popliteal Lymphnode ^2^	Peripheral Blood ^3^	Conjunctival Swab ^4^
Group 1	22.4 (20.3–28.6) ^a^	22.9 (17.8–36.6)	N/D ^a^	30.3 (27.1-ND) ^a^
Group 2	ND (18.2-ND) ^b^	25.6 (17.5-ND)	ND (32.2-ND) ^a^	30.4 (26.4-ND) ^a^
Group 3	24.2 (17.2–28.2) ^a^	26.1 (21.2–27.1)	ND (31.3-ND) ^b^	27 (24-ND) ^b^

^a,b^ Superscripts with different letters in the same column show significant differences between groups. ^1^ Kruskal-Wallis *p*-value (P_K-W_) = 0.025; ^2^ P_K-W_ = 0.246; ^3^ P_K-W_ = 0.029; ^4^ P_K-W_ = 0.041; ND: Ct ≥ 40.

**Table 4:** Sybr-qPCR results in the different biological samples studied according to clinical signs classification.

	Bone Marrow ^1^	Popliteal Lymph Node ^2^	Peripheral Blood ^3^	Conjunctival Swab ^4^
Group 1	23.1 (19.5–28.7) ^a^	23.2 (19.1–25.4)	33.8 (32.1–35)	32.1 (27.8–35.3) ^a^
Group 2	25.9 (20.8–33.9) ^b^	25.7 (21–33.6)	33.1 (32.3–35.4)	31.7 (27.28–35.1) ^a^
Group 3	24.7 (17.7–28.2)	27.4 (22.2–29.7)	32.4 (27.4–35.6)	28.1 (24.1–33.2) ^b^

^a,b^ Superscripts with different letters in the same column show significant differences between groups. ^1^ P_K-W_ = 0.016; ^2^ P_K-W_ = 0.051; ^3^ P_K-W_ = 0.603; ^4^ P_K-W_ = 0.048.

**Table 5:** TaqMan-qPCR results in the different biological samples studied according to serological evaluation.

Technique	Result	Bone Marrow ^1^	Popliteal Lymph Node ^2^	Peripheral Blood ^3^	Conjunctival Swab ^4^
DAT	Positive	25.2 (18.2-ND)	25.2 (21.2–27.4)	32.2 (ND-ND)	28.2 (24-ND)
	Doubtful	22.6 (17.2–26.7) ^a^	22.9 (17.8–26.1)	ND (31.3-ND)	29.9 (27-ND)
	Negative	ND (20-ND) ^b^	32.1 (17.5-ND)	ND (32.2-ND)	36.2 (24.2-ND)
IFA	Positive	24.1 (17.2-ND) ^a^	24.4 (17.8-ND)	31.38 (ND-ND)	29.2 (24-ND)
	Doubtful	ND (28.6-ND) ^b^	36.2 (17.5-ND)	ND (32.2-ND)	ND (28.4-ND)
	Negative	ND	ND	ND	ND
ELISA	Positive	24.1 (17.2-ND) ^a^	24.7 (17.8-ND)	ND (31.3-ND)	29.2 (24-ND) ^a^
	Doubtful	-	-	-	-
	Negative	ND (26.7-ND) ^b^	33.9 17.5-ND)	ND (32.2-ND)	36.2 (28.4-ND) ^b^

^a,b^ Superscripts with different letters in the same column show significant differences between groups in each technique. ^1^ DAT P_KW_ = 0.013; IFA P_KW_ = 0.006; ELISA Mann-Whitney *p*-value (P_MW)_ = 0.006. ^2^ DAT P_KW_ = 0.204; IFA P_KW_ = 0.22; ELISA P_MW_ = 0.082. ^3^ DAT P_KW_ = 0.38; IFA P_KW_ = 0.739; ELISA P_MW_ = 0.086. ^4^ DAT P_KW_ = 0.4; IFA P_KW_ = 0.074; ELISA P_MW_ = 0.097.

**Table 6:** Sybr-qPCR results in the different biological samples studied according to serological evaluation.

Technique	Result	Bone Marrow ^1^	Popliteal Lymph Node ^2^	Peripheral Blood ^3^	Conjunctival Swab ^4^
DAT	Positive	24.7 (20.8–32.2)	26.4 (22.4–29.9)	34.3 (32.2–35.6)	28.8 (26.2–34.2)
	Doubtful	23.4 (17.7–25.9) ^a^	24 (19.1–28.2)	33.1 (27.4–35)	31.07 (27.8–33.6)
	Negative	29.8 (20.2–33.9) ^b^	33 (32.4–35.4)	25.9 (21–33.6)	32.24 (24.1–35.3)
IFA	Positive	23.9 (17.7–32.2) ^a^	24.8 (19.1–29.9)	33.2 (27.4–35.6)	30.4 (24.1–34.2)
	Doubtful	31.7 (25.4–32.7) ^b^	29.7 (21–33.6)	33 (32.5–34.1)	33.5 (29.1–35.3)
	Negative	33.9 (33.9–33.9)	33.2 (33.2–33.2)	33.4 (33.4–33.4)	33 (33 -33)
ELISA	Positive	23.9 (17.7–32.2) ^a^	24.8 (19.1–29.9)	33.1 (27.4–35.6)	30.4 (24.1–35.3)
	Doubtful	-	-	-	-
	Negative	31.8 (24.9–33.9) ^b^	30.4 (21–33.6)	33.2 (32.5–35)	32.5 (29.1- 35.1)

^a,b^ Superscripts with different letters in the same column show significant differences between groups in each technique. ^1^ DAT P_KW_ = 0.013; IFA P_KW_ = 0.006; ELISA P_MW_ = 0.006. ^2^ DAT P_KW_ = 0.204; IFA P_KW_ = 0.22; ELISA P_MW_ = 0.082. ^3^ DAT P_KW_ = 0.38; IFA P_KW_ = 0.739; ELISA P_MW_ = 0.086. ^4^ DAT P_KW_ = 0.4; IFA P_KW_ = 0.074; ELISA P_MW_ = 0.097.
